# Development of a Web-Based Decision Aid and Planning Tool for Family Building After Cancer (Roadmap to Parenthood): Usability Testing

**DOI:** 10.2196/33304

**Published:** 2022-05-31

**Authors:** Catherine Benedict, Katherine L Dauber-Decker, Jennifer S Ford, D'Arcy King, David Spiegel, Lidia Schapira, Pamela Simon, Michael Diefenbach

**Affiliations:** 1 Stanford University School of Medicine Stanford, CA United States; 2 Stanford Cancer Institute Palo Alto, CA United States; 3 Donald and Barbara Zucker School of Medicine at Hofstra/Northwell Manhasset, NY United States; 4 Hunter College and The Graduate Center City University of New York New York, NY United States; 5 Lucile Packard Children’s Hospital Stanford Palo Alto, CA United States

**Keywords:** young adult cancer, cancer survivorship, decision-making, decision aids, fertility, reproductive health, mobile phone

## Abstract

**Background:**

Owing to gonadotoxic cancer treatments, young adult female survivors often report uncertainty about their fertility, reproductive potential, and family-building options after treatment. *Roadmap to Parenthood* is a web-based decision aid and planning tool for family building after cancer.

**Objective:**

As part of a patient-centered development process, this study evaluated the usability of the decision aid website to inform design modifications and improve user experience.

**Methods:**

In total, 2 rounds of usability testing were conducted with the target population of young adult female cancer survivors. During the testing sessions, participants viewed the website twice; first, as a *think-aloud* exercise, and second, while a researcher interrupted at key points to obtain user feedback. Quantitative and qualitative data were collected to assess website usability. Quantitative measures included the System Usability Scale, WebQual, and eHealth Impact Questionnaire. An exit interview with open-ended questions gathered feedback on likes and dislikes and suggestions for improvement.

**Results:**

Participants (N=10) were young adult women, with average age of 30.9 (SD 4.51) years, and average time since treatment was 4.44 (SD 3.56) years. Website usability scores improved on the System Usability Scale from “acceptable” in round 1 to “excellent” in round 2 after making design changes based on user feedback (scores of 68 and 89.4, respectively). WebQual scores showed similar improvement from round 1 to round 2 of testing (mean 5.6 to 6.25; range 1-7). On the eHealth Impact Questionnaire, the information and presentation of the website was perceived as comprehensive, easy to understand, and trustworthy. Participants also reported improved confidence to discuss and manage fertility and family-building issues and felt encouraged to play a more active role in managing their fertility. In all, 3 usability themes were identified from the qualitative feedback: ease of use, visibility and navigation, and informational content and usefulness. Overall feedback was positive, and participants reported intentions to use the decision aid website in the future. In total, 10% (1/10) of the participants reported negative emotions when learning about infertility risks and potential family-building challenges.

**Conclusions:**

Website usability improved after design changes were made in response to user feedback. Young adult female survivors reported positive views about the website and indicated that the decision aid would be useful in decision-making about family building after cancer. Future studies will include further design modifications to consider the emotional experiences of users and any additional navigational features or content to optimize the ease of use and support provided by the tool.

## Introduction

### Background

In young adult cancer survivors, gonadotoxic treatments may negatively affect fertility and reproductive health [1,2]. In the aftermath of cancer, questions surrounding fertility status and implications for family-building options are often distressing, particularly for young women who may wonder about their chances of achieving pregnancy, reproductive time line, or health risks [3,4]. On the basis of the principles of patient-centered care, women should be supported in making informed, values-based decisions that align with their long-term goals for family building [5]. Resources are needed to educate and support women in seeking reproductive health care after treatment and making decisions about family-building options.

### Fertility and Family Building After Cancer

It is well established that young adult female cancer survivors are often uninformed and worried about potential fertility issues following cancer treatment and endorse high rates of unmet support needs [6-8]. Most are unable to preserve fertility before treatment owing to many factors (eg, time constraints, emotional distress, and cost), and there is great uncertainty about fertility and family-building options after treatment is completed [9-11]. Among women who wanted children after cancer, 64% worried about fertility problems; however, only 10% had undergone fertility evaluation since their treatment ended [6]. Fertility is recognized as an important survivorship issue [12]; however, patients are often not counseled about options to evaluate and monitor fertility over time or about alternative family-building options if natural conception is not possible. These options include using reproductive medicine (eg, in vitro fertilization or surrogacy with fresh, frozen, or donated gametes) or adoption or fostering, but have medical, psychosocial, financial, legal, and logistical challenges [13-15].

### Decision-making About Family Building After Cancer

Making decisions about reproductive health care and family building after cancer can be overwhelming and distressing. Previously, we found high rates of decisional conflict about family building among young adult female survivors who reported feeling uninformed about their options (86%) and unclear about personal values (74%) and lacked guidance (70%) and adequate emotional support (35%) [6]. Even when informed, women still face uncertainties surrounding inexact estimates of fertility potential, likelihood of success with assisted reproductive technology, health risks, direct and indirect costs, and unknown bureaucratic difficulties. Decision aids have proven effective in helping young women diagnosed with cancer to make decisions about fertility preservation before treatment [16]. We are aware of 10 decision aids designed for women with cancer who are considering fertility preservation before treatment (only 4 aids are in English and 6 aids are for breast cancer only); efficacy data are available for 4 of these decision aids as of September 2021. Studies report good acceptability and satisfaction among women and positive effects on decision-making outcomes (eg, improved knowledge and decisional conflict) [17-19]. Consistent with the broader decision science literature [20], these studies provide initial support for the utility of decision aids for young adult female cancer survivors facing fertility decisions. However, none of them include comprehensive information about decisions that must be made *after* treatment is completed to address follow-up questions about fertility outcomes and decisions about reproductive health care and family building and to help plan for the future for those not yet ready to start their family building. Other oncofertility resources exist (eg, educational materials), but these are of varying quality with limited data about their development and efficacy; very few describe user-centered design processes [21].

On the basis of extensive pilot study and following user-centered design practices [6,11,13,22], we developed a web-based decision aid and planning tool for family building after cancer, *Roadmap to Parenthood*. Briefly, the interactive tool provides information about cancer treatment’s effects on fertility and family-building options if natural conception is not possible and includes a values-clarification tool, family-building stories from other survivors, and guidance for *next steps* action planning. Additional resources include in-depth information about specific topics, financial loans and grants, and psychological support including connecting with cancer-related and fertility-related organizations. It was designed to be used by single and partnered women, inclusive of sexual orientations, and it is appropriate for all stages of decision-making readiness and expected family-building time lines. In other words, women can use the tool to make intermediary decisions about preparatory actions (eg, seek a fertility evaluation, undergo fertility preservation after treatment if possible, or plan financially) and plan for the future if they are at risk for experiencing fertility problems (eg, premature menopause), but their desired time frame for parenthood is not many years. Ultimately, the overarching goal of the *Roadmap to Parenthood* decision aid tool is to encourage survivors to be informed about family-building options, set realistic expectations about potential difficulties, and plan ahead if desired, while also inspiring hope and confidence that parenthood may be achieved, despite their cancer histories.

### Objectives

Previously, we described the development of the *Roadmap to Parenthood* decision aid website prototype [23]. Previous studies have shown that usability testing can help developers optimize decision support tools for future end users [24,25]. The usability of such a tool refers to the extent to which it may be used effectively and efficiently to achieve specified goals in a specified context of use and includes user satisfaction (eg, the tool is easy to learn, tasks can be performed quickly with minimal errors, and the design is pleasant) [26,27]. Usability is an aspect of the overall user experience, which is a broad concept that includes all components of a user’s motivations and needs and their interaction with and perceptions of the tool, such as whether it is useful, usable, desirable, accessible, credible, and valuable [28,29]. Here, we have reported the results of usability testing of the tool and responsive design changes as part of an iterative user-centered development process. Our goal was to evaluate and improve the usability of the website, thus contributing to a positive user experience, and to optimize the website as a support resource for young adult female cancer survivors.

## Methods

The study was conducted at Northwell Health and the affiliated Feinstein Institutes for Medical Research, a large academic hospital and research institute in New York.

### Ethics Approval

All the study procedures were approved by the Northwell Health Institutional Review Board (18-0516).

### Decision Aid Prototype

**Figure 1:**
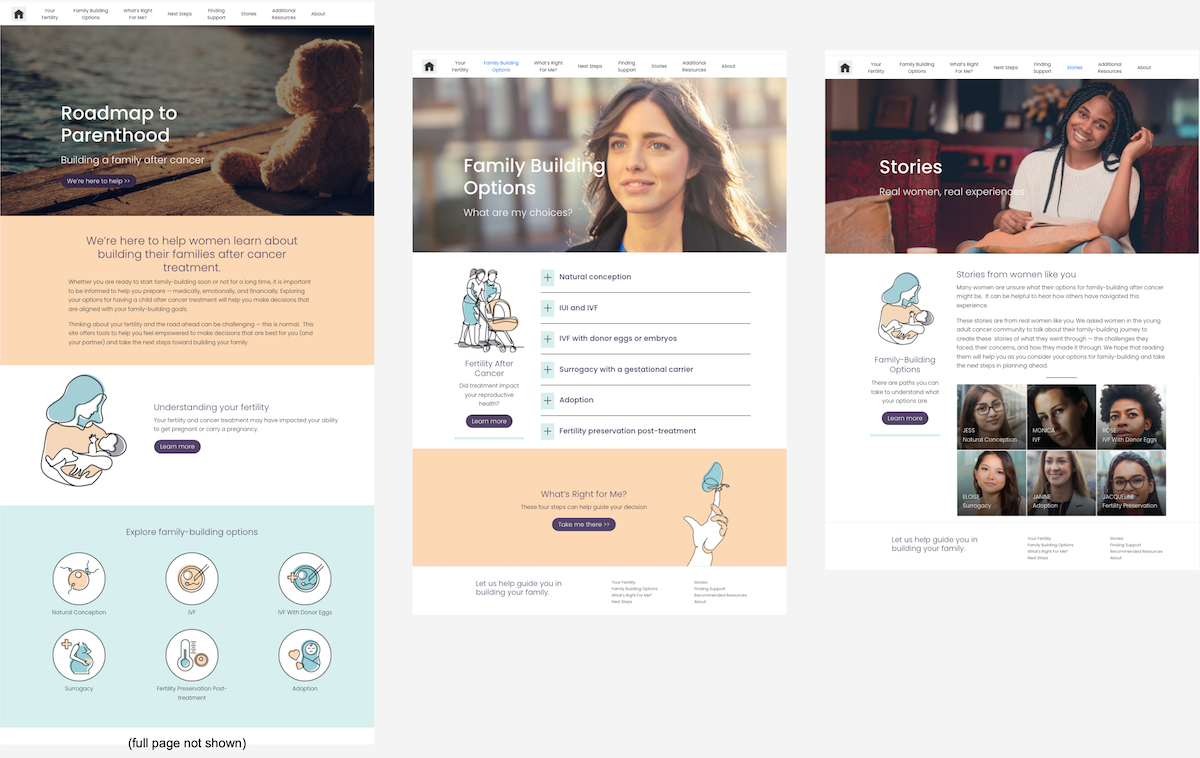
Selected pages from the Roadmap to Parenthood decision aid website. Some of the design aspects illustrated by these pages include the top and bottom navigation bars; using color, white space, and drawer design to chunk and divide sections; using icons to indicate information about family-building options; and call-to-action buttons (side and bottom) to guide the user journey.

As described in the previous section, the *Roadmap to Parenthood* is a web-based decision aid and planning tool for family building after cancer, designed to be used by young adult female cancer survivors who may be at risk for fertility and family-building problems owing to gonadotoxic treatments. It is based on the experiences of young adult female survivors in the United States and is written in English. Personalized information about infertility risk and likelihood of success with family-building options is not provided. The design of the decision aid followed guidelines from the International Patient Decision Aid Society and Ottawa Decision Support Framework for developing patient decision aids [30-32]. Guidelines from the Office of Disease Prevention and Health Promotion [33], National Institutes of Health [34], Centers for Medicare and Medicaid Services [35], and Stanford University Office of Digital Accessibility [36] also were followed to ensure that the design and content were accessible to most users, including users with varying levels of health literacy, users with disabilities, and culturally diverse populations (previously described by Benedict et al [23]). Selected pages from the website are shown in Figure 1.

### Participants

Eligible participants were English-speaking women, aged 18 to 39 years, who completed potentially gonadotoxic cancer treatment (eg, systemic chemotherapy, radiation to the pelvic area or brain, or surgery affecting the reproductive organs) and reported a desire for future children or uncertainty about family-building plans. Participants needed to have internet access and use a computer, tablet, or smartphone.

### Procedures

Participants (N=10) were recruited through the Northwell Health system and social media. It has been established that testing a product with 5 participants is sufficient to reveal approximately 80% of the product’s usability issues [37]. For hospital-based recruitment, a list of eligible patients was generated using electronic health record data, and letters were mailed to invite participation. Young adult cancer organizations (eg, Stupid Cancer and Lacuna Loft) also posted institutional review board–approved advertisements on their social media pages, with links to submit contact information via a secure platform. A research coordinator followed up with those who submitted their contact information via a telephone call to describe the study and answer questions. Participants were provided with information about the study objectives and participation requirements. Written informed consent was obtained remotely (via REDCap [Research Electronic Data Capture; Vanderbilt University]), and then, the participants were scheduled for a usability testing session. Compensation (US $10) was provided after completion of the testing session.

Usability testing was conducted by the principal investigator (PI) and Northwell Usability Lab, which has extensive experience and expertise in developing patient decision aids following user-centered design approaches, including usability testing and data analysis [38]. Totally, 2 rounds of usability testing were conducted, allowing the study team to make design changes that were responsive to initial usability problems and, then, to test an updated prototype. Each participant completed only 1 usability testing session (ie, different participants were included in round 1 and round 2 of testing). The first round of testing included 6 testing sessions, at which point saturation was reached in identifying usability issues [39], and a decision to halt testing to address critical design flaws was made. Following changes to the website in response to the initial user feedback, the second round of testing included 4 additional sessions with new participants to evaluate the updated website.

For each round of usability testing, participants completed a brief baseline survey and then participated in a usability testing session (30-45 minutes). A member of the Northwell Usability Lab led the testing sessions with at least one other study team member and the PI (CB) present, allowing for observer triangulation to identify problems in user experience and design. To evaluate website usability, certain key issues were assessed, including evaluation of users’ ability to find desired information, evaluation of the clarity of and comfort with the website content, and identification of barriers to full use and ease of navigating through the website. Participants were asked to view the website prototype twice. First, they explored the website in a *think-aloud* exercise in which they provided a verbal talk-track describing their experiences with the content and navigation through the site, while the researchers also observed how they interacted with the site. Then, they reviewed the website a second time, and a researcher interrupted at key points to obtain feedback on specific visual and written content, transitions between webpages, information flow, and design. These interruptions were responsive to the actions of the users as they explored the website and enabled the research team to clarify various aspects of the user experience, points of confusion, and users’ preferred modifications. The sessions were recorded via audio and screen capture (ie, recording participants’ verbal feedback and visual representations of website navigation), and members of the research team took notes during each testing session. Upon completion, participants completed a survey and an exit interview.

### Measures

The baseline survey included standard sociodemographic and medical history questions and the *eHealth Impact Questionnaire (eHIQ)-part 1* (10 items), a validated measure of general attitudes toward using the internet to access health information and perceived value of web-based health-related resources [40].

Following the testing session, several measures quantified the usability and impact of the website, including aspects of user experience (eg, perceptions of credibility and value). The *System Usability Scale* (SUS; 10 items) is a reliable, industry-standard tool to measure perceived ease of use of a website across usability factors [41]. Scores are converted to a 0 to 100 scale, with a score of 68 considered as cutoff point for “above average” and a score of 85 considered as “excellent” usability [42,43]. *WebQual* (7 items) is a multidimensional measure of consumer evaluation of websites (eg, perceived usefulness, ease of use, and intent to reuse the website) [44]. Scores range from 1 to 7, with higher scores indicating more positive evaluation of the website. The eHIQ-part 2 assesses the impact of using a specific website for health purposes. Subscales of the eHIQ-part 2 include the following: *Confidence and Identification*, measuring confidence to discuss health with others and ability to identify with the website (9 items); *Information and Presentation*, measuring perceived trust and suitability of the website content (8 items); and *Understanding and Motivation*, assessing understanding and learning about relevant information and motivation to take action (9 items) [40]. Scores range from 0 to 100, with higher scores indicating more positive evaluation and impact of using the website. Finally, open-ended questions during the exit interview explored the participants’ overall impressions, likes and dislikes, emotional reflections, recommendations, and expectations for future use.

### Data Analysis

Descriptive statistics were used to analyze the sociodemographic and medical characteristics of the sample and the eHIQ-part 1 data, providing a baseline understanding of participants’ general attitudes toward web-based health resources (ie, not specifically related to the decision aid tool).

Qualitative and quantitative data from the usability testing sessions were analyzed. Think-aloud feedback and answers to the open-ended questions of the exit interview were analyzed qualitatively to capture perceptions of usability, aspects of user experience, and user recommendations for design changes. Coding team members performed content analyses of testing session notes, grouping the feedback points from each participant into overarching categories based on *a priori* codes derived from previous studies on developing patient decision aids and the literature [25,45]. An iterative process of coding and group discussion was conducted to verify initial codes, definitions, and overarching themes. At least 2 team members coded all the data. Team members revisited the audio and Hypercam (Hyperionics) recordings for content and wording clarifications when necessary. Northwell Usability Lab members ensured that coding was consistent across coders by creating a code book with definitions, discussing how they would code sample sections of the session notes and confirming team member agreement of coded data. The PI (CB) reviewed the coding and thematic categories and discussed with the team how the results should be used to make website design changes. Data from open-ended questions during the exit interview were summarized to identify patterns in likes and dislikes, emotional reflections, and recommendations and for additional context to understand participant feedback and the overall user experience. In addition, quantitative survey data (ie, SUS, WebQual, and eHIQ-part 2) collected after participants completed the usability testing session were summarized descriptively. Data were divided between the 2 rounds of testing (round 1: 6/10, 60% of the total sample and round 2: 4/10, 40% of the total sample) and compared. Design modifications were made after the first round of testing, when content analysis of the testing data indicated that no new usability issues were identified. Thus, comparing the results across these subgroups allowed us to evaluate whether improvements in usability were successful with design changes and assess whether optimal usability had been reached. Given the small sample size, tests of statistical significance were not performed.

## Results

### Participant Characteristics

Participants (N=10) were young adult female cancer survivors with average age of 30.78 (SD 4.51) years, with previous diagnoses of breast cancer, cervical cancer, uterine or endometrial cancer, Hodgkin lymphoma, or leukemia. On average, time since treatment was 4.44 years (SD 3.56 years; Table 1).

At baseline, before viewing the website, participants reported strong agreement that the internet can be useful to deal with health problems, and 60% (6/10) of the participants *agreed* or *strongly agreed* that they would use the internet to help make decisions about health (eHIQ-part 1 items). The internet was also seen as a good resource to learn about others’ health-related experiences and decision-making, and health-related websites could provide reassurance that participants were not alone with their health concerns.

**Table 1:** Sociodemographic and medical characteristics of the sample (N=10).

Sample characteristics		Values
Age (years), mean (SD; range)		30.90 (4.51; 25-39)
Age at diagnosis (years), mean (SD; range)		26.13 (6.59; 15-35)
Time since treatment (years), mean (SD; range)		4.44 (3.56; 0.6-10.92)
**Race, n (%)**		
	White	9 (90)
	>1 race	1 (10)
**Ethnicity, n (%)**		
	Hispanic	1 (10)
**Education (highest attained to date), n (%)**		
	High school degree	1 (10)
	College degree	4 (40)
	Postgraduate degree	5 (50)
**Cancer diagnosis, n (%)**		
	Breast	2 (20)
	Cervical	1 (10)
	Uterine or endometrial	1 (10)
	Hodgkin lymphoma	5 (50)
	Leukemia	1 (10)
**Cancer treatment (not mutually exclusive), n (%)**		
	Chemotherapy	10 (100)
	Surgery that involved removal of the uterus or both ovaries	2 (20)
	Radiation that included the abdominal or pelvic region or brain	3 (30)
	Bone marrow or stem cell transplant	2 (20)
	Hormone therapy or immunotherapy	1 (10)
	Other	1 (10)

### Usability Survey Data

**Figure 2:**
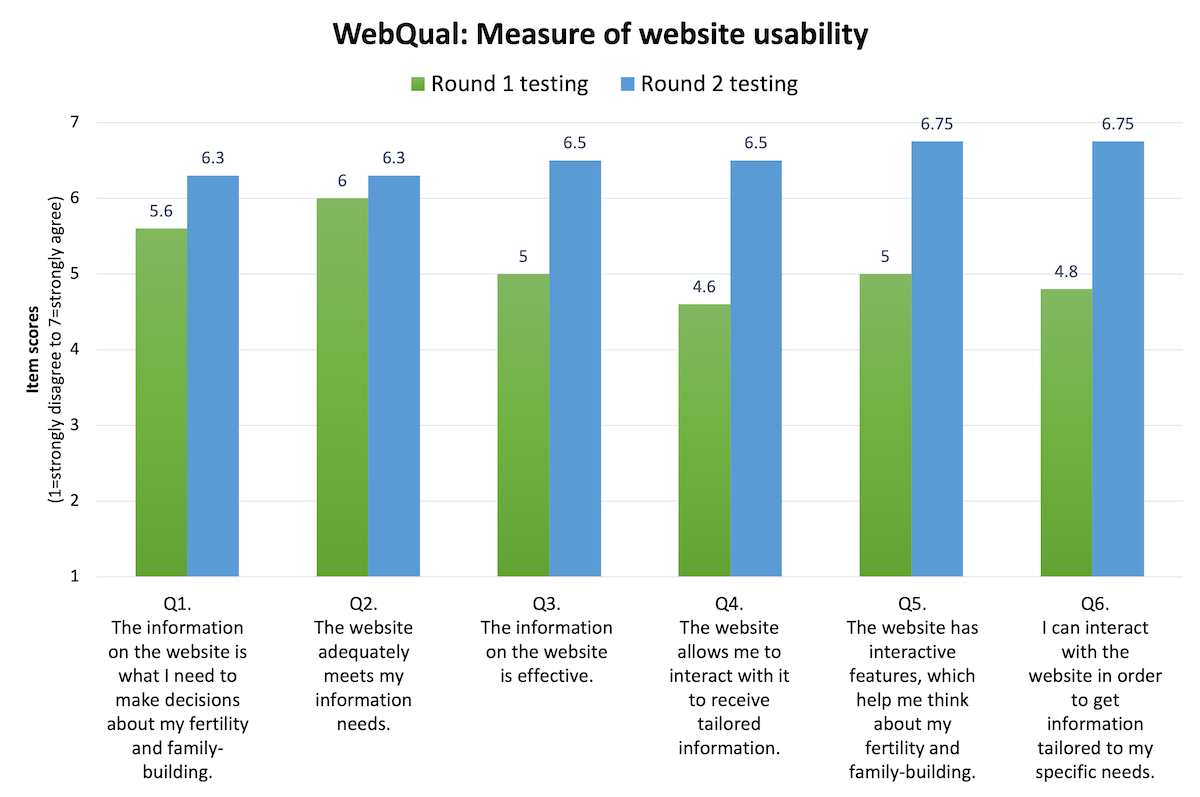
WebQual: Measures of website usability. Improvements in website usability were observed from round 1 to round 2 of testing (pre-post design changes). Average scores across WebQual items are depicted for both rounds of testing. The possible range of scores is 1 (strongly disagree) to 7 (strongly agree).

Scores on usability measures demonstrated improvement in website usability from round 1 to round 2 of testing (before and after making design changes). SUS scores in round 1 averaged 68 (possible range 0-100), indicating “acceptable” usability. After design modifications, SUS scores in round 2 averaged 89.4, representing “excellent” usability and reaching the threshold for optimal usability for a website. Average WebQual scores (possible range 1-7) also improved from round 1 (mean 5.6) to round 2 of testing (mean 6.25; Figure 2). On a scale from 1 (not at all likely) to 10 (extremely likely), all participants in round 2 responded that they would recommend the website to a friend or other cancer survivor, with scores ranging from 9 to 10.

**Figure 3:**
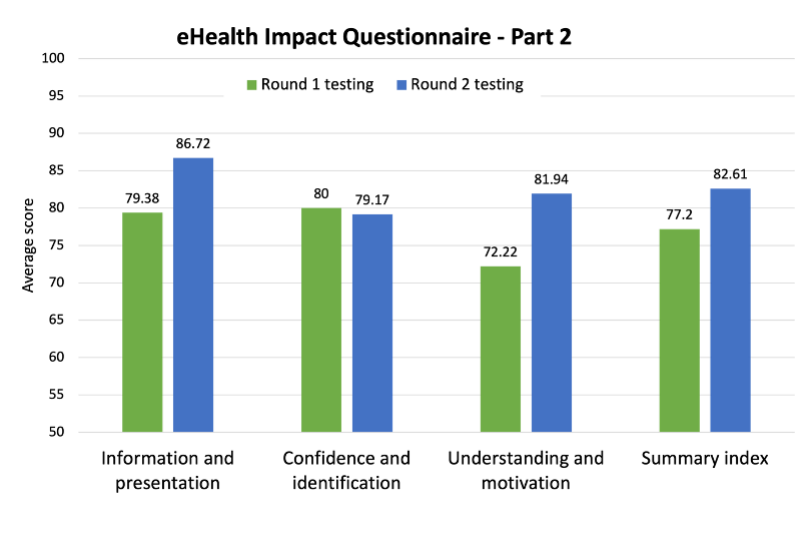
eHealth Impact Questionnaire - Part 2. Improvements were observed across 2 of the 3 domains of the eHIQ-part 2, assessing the impact of using the *Roadmap to Parenthood* website.

Perceptions of the website were also evaluated using the eHIQ-part 2. Previous studies have used a cutoff score of ≥65 for eHIQ subscales to indicate that the website was rated positively by users [46], and all subscale scores in round 2 of testing were higher than this cutoff (Figure 3). The information in and presentation of the website were perceived as being comprehensive and easy to understand, and pictures or images were viewed as being used appropriately. In addition, the website was perceived as trustworthy. Participants reported that the website improved their confidence to discuss fertility and family-building topics with others and to manage difficulties that may arise, while also indicating that they identified with other people who use the website. Participants reported that the website felt reassuring, helped them gain a better understanding of their fertility and family-building options, and encouraged them to play a more active role in managing their fertility to align with their family-building goals. All participants indicated that they agree or strongly agree with the statements, “the website encourages me to take actions that could be beneficial to my health” and “I feel more inclined to look after myself after visiting the website.” 

### Qualitative Feedback

#### Overview

Data from the think-aloud portion of the usability testing sessions were analyzed to further characterize the usability issues. Totally, 3 main themes that represent different aspects of website usability were identified: ease of use, visibility and navigation, and informational content and usefulness. Table 2 describes the main themes identified across the 2 rounds of testing and the content modifications and design solutions that were implemented after round 1 or are planned to be implemented in response to round 2 feedback. Consistent with survey data, we observed differences in qualitative feedback, suggesting that the initial design problems identified in round 1 were largely resolved with the modifications that were made. In contrast, round 2 of testing identified more specific and nuanced usability problems and patient-driven suggestions for design changes.

**Table 2:** Qualitative themes identified from usability testing.

Theme	Definition	Sample quotes	Design changes
Ease of use	Describes how easily users could use and understand the website	“I like the family building options section a lot – a nice menu showing all the options and then you can click on the one you want to learn more about – sticks out as being very user-friendly” “...just the presentation of the data. Once I knew what my options were, I would want a lot of information. But I would only want to see that about what I was feeling right then and get the information about that one thing. Bullet points.”	Attention to health literacy and reading levels, for example, use of medical terminology with definitions Improved presentation of information, for example, better use of headers and subheaders, font and color changes, short paragraphs, white space, and use of drawer design Language or word changes to improve relatability
Visibility and navigation	Website workflow: whether the website’s components were readily and easily discoverable (visible) and whether users found it easy to transition between different parts of the website in a logical and intuitive manner (navigate)	“I feel like when I go from page to page, it flows really well, but I feel like I’m so deep into it that when I want to go to a different page or the beginning, I don’t know where to go. The restart is very helpful. But I do feel like there’s a logical flow otherwise.”	Top navigation bar and drop-down menus depicting website pages Call-to-action buttons In-page guidance (signposts) suggesting next steps in the user journey Navigation footer (particularly helpful for mobile phone users)
Informational content and usefulness	Presentation of information and the utility of informational content	“I like [the personal worksheet page] because it’s interactive in a way that what you’re reading from the website, you can use on this and it’ll help you take the next step.” “This is really interesting to me – very applicable to my stage of family building. I had no idea how expensive it was going to be to build a family afterwards. I felt like everybody said, ‘oh freeze your eggs!’ but they never said how expensive it would be after, and now I feel pressure having the eggs and having to take that path.” “Maybe it’s just [because] that’s something that’s a really big fear for a lot of women, but [the graph of declining ovarian reserve] is terrifying. I don’t know that that would be the first thing I wanted to see if I went here because that’s where everybody’s mind is going to go immediately is like, ‘Oh no, what if this happens?’...It might be scary.”	Reordering of content with careful consideration of text and images Changes to highlight important pieces of information in conjunction with images or pictures Improved user journey and navigation guide to connect information to appropriate follow-up pages and resources for support (eg, in-page links and signposts to connect user to resources)

#### Ease of Use

The *ease of use* theme describes the degree of ease with which users can use and understand the website. Round 1 of testing identified several significant problems that affected the website’s ease of use and the overall user experience. Participants felt that the presentation of information was overwhelming in sections that had long paragraphs of text and on pages that required excessive scrolling to view the content. Accordingly, for pages containing large amounts of content, a *drawer design* was implemented such that the content was hidden from view and only made visible if the user clicked on the header (Figure 1), or the content was divided into separate pages. In addition, font sizes and colors were changed to make the section headers more noticeable, divide the text more clearly, and use white space effectively. Text was divided into short, more digestible paragraph lengths to aid both readability and comprehension.

Compared with round 1, comments obtained in round 2 were more specific. Some participants pointed out preferences around syntax that interfered with optimal use of the website, mentioning specific words or phrases they disliked or found unclear or suggesting optimizations with the content.

#### Visibility and Navigation

The *visibility and navigation* theme focuses on feedback on the website’s workflow, including whether the website’s components are readily and easily discoverable (visible) and whether users find it easy to transition between different parts of the website in a logical and intuitive manner (navigate). In round 1, participants had difficulty in navigating through the website pages (eg, difficulty in finding pages, got lost between pages, and dead-end pages). The website was designed to personalize their user journey, allowing them to access relevant content based on their needs and where they were in their family-building time line. This involved an omnichannel user journey design, similar to *choose your own adventure*, in which users could follow any number of user pathways specific to their needs. Initial testing in round 1 revealed errors in the user journey flow and a lack of structure in guiding the user experience, which appeared to be confusing to the user. To address these issues, navigation bars and call-to-action buttons were added to help signpost content across website pages and guide the user journey. Drop-down menus were also added to the navigation bar to depict multiple pages within the same section, allowing users to jump to the section they were most interested in, while maintaining visual cues to easily move to the other sections as desired. For example, the *Next Steps* tab of the top navigation bar had a drop-down menu including each page within that section (eg, *Ask Your Oncologist, Ask a Fertility Specialist, Financial Planning*, and *Talk to Your Partner*). In addition, callout links were added to help with navigation by suggesting next pages to visit, thereby providing guidance for the user journey, while simultaneously providing users the freedom to bypass the callouts and follow their preferred path if desired.

The visibility and navigation issues identified in round 1 appeared to be largely resolved in round 2 of testing with the design changes that were implemented. However, additional minor problems were identified, which will be addressed in the next iteration of the decision aid website.

#### Informational Content and Usefulness

The *informational content and usefulness* theme emphasizes how information is presented on the website and the utility of informational content to future end users. In round 1 of testing, some of the most poignant feedback was given in response to the *Understanding Your Fertility* page, which provided information in the form of text and graphs about female reproductive health and potential effects of cancer treatment on fertility. A user found the graphs of declining ovarian reserve with advancing age and impact of cancer treatment “terrifying” and suggested changing the order of the graph and text to reduce the emotional impact. We made the suggested changes and modified the text to more clearly highlight that the data presented are based on population-level statistics and may not apply to all women and that users must speak with a health care provider to obtain individualized information about their reproductive health. The graph was also edited to soften the depiction of risks surrounding infertility and appear less threatening (eg, bold cutoff points were changed to gradations). A few participants also suggested ways to improve the relatability of the site. For example, a user noted that some survivors want children but do not identify as “young”; therefore, the use of this terminology could make the website feel less relatable. An additional area where users felt content and usefulness could be improved was the *Personal Worksheet* page. A participant suggested that we add more detailed information on next steps based on the users’ worksheet data. Other suggestions indicated a need for more information on early menopause, specific questions to ask a fertility specialist, and more direct links to external resources and organizations that users could access in the future. Content was edited in response to each comment that was received.

After we made modifications based on round 1 feedback, participants of round 2 had suggestions for additional helpful content focused on financial information, insurance coverage limitations, finding adoption agencies that work with cancer survivors, working with surrogate agencies or attorneys, contacting human resource departments for assistance, and information about genetic risks.

#### General Feedback

Finally, the exit interviews provided additional data about usability and user experiences and included general feedback about users’ likes and dislikes, emotional reflections, and recommendations. Overall, we received positive feedback from participants about the website. Young adult women reported that they identified with the website and felt the information was relevant to their needs:

You understand me as a woman really well. You understand what kind of information I’d be looking for.

When asked what they liked best about the website, the most common answer was the inclusion of stories representing family-building options, with participants stating that it felt good to hear peer stories with which they could identify. Other sections noted as favorites were those providing information about talking to one’s partner, questions to ask one’s oncologist, the values-clarification exercise, and the resources page. Participants reported that they liked the breadth of information and felt it was relevant and accessible. When asked what they liked the least about the website, consistent with qualitative themes, participants indicated navigation problems (primarily in round 1) and other minor design and content issues (eg, small font size). When asked if anything was missing from the website, participants indicated a preference for more photographs and videos and again suggested additional information topics and resources (eg, app recommendations for period tracking). Participants described the website as a “unique resource” and “one-stop-shop tool to learn about fertility options and to help you make informed decisions.” They indicated that the information was comprehensive and understandable:

For normal people...not too scientific, but for people like us.

They also discussed the emotional impact of having access to the decision aid tool:

A really great resource depending on your own individual situation to make you less overwhelmed and guide you through the process.

When prompted for final thoughts and impressions, a participant said the following:

Let me know when I can share it with the world [because] I know a lot of people that would find this helpful.

Overall, this positive feedback was encouraging and suggested that the decision aid tool would be useful, appealing, and well received by future end users.

## Discussion

### Principal Findings

The aim of this study was to evaluate the usability of a web-based decision aid and planning tool for family building after cancer, *Roadmap to Parenthood*, to inform design modifications and better understand user experience as part of an iterative, user-centered development process. Website usability was evaluated quantitatively and qualitatively across 2 rounds of testing, along with some aspects of user experience, to understand the context and impact of using the decision aid tool. Average usability scores improved from “acceptable” in round 1 to “excellent” in round 2 after making design changes based on user feedback. We identified 3 usability themes that represented issues related to ease of use, visibility and navigation, and informational content and usefulness. This study is among the limited number of usability studies that evaluated digital health tools for young adults affected by cancer and, to the best of our knowledge, the only evidence-based decision support resource for family building after cancer [21,47].

Website usability improved with modifications based on initial user feedback, including user perceptions of how easy it was to use the website, find information, and navigate through the website and their perceptions of its content and usefulness. However, as just a part of the user experience, the broader context must also be understood. User experience includes the motivations, emotions, and expectations that users have before interacting with the technology; end-to-end interaction with the technology; and reflective emotions and behaviors after use. At baseline, this sample of young adult cancer survivors had positive views about using the internet for health-related problems, including to access information and support for health-related decision-making. General feedback about the website was positive, and users reported an intention to use the decision aid in the future. All participants, across both rounds of testing, *agreed* or *strongly agreed* that they felt more informed after viewing the website and would consult the website to make decisions about fertility and family building. They trusted the information on the website and felt understood by the people who developed it. In open-ended feedback, participants consistently expressed appreciation that a trusted resource existed, as they were otherwise unsure about where to access this information and decision support. In a systematic review of web-based oncofertility decision aids and health education materials, the quality of websites was found to be variable and, among the decision aids, the content focused primarily on fertility preservation before cancer treatment initiation [21]. More generally, public websites providing cancer-related information have been shown to be largely incomplete in the information they provide, with questions about decision-making being discussed the least [48]. Furthermore, web-based patient information about cancer survivorship and fertility preservation has been shown to be written, on average, at high school senior and junior college levels [49], thus failing to meet health literacy standards [50]. Results suggest that finding reliable, understandable, and trustworthy information about family building after cancer may be a difficult task for young adult female cancer survivors and this decision aid tool fills this critical unmet need.

Unlike most decision aids that are developed for one-time treatment decisions involving discrete time [20], in this case, decision making may include an ongoing process of considering numerous intermediary decisions along the path to family building, such as considering a reproductive endocrinologist consultation, seeking legal advice, and looking for financial planning information. Women may also need to reconsider decisions if their preferred option to achieve parenthood is unsuccessful, such as considering donated gametes, surrogacy, or adoption after failed in vitro fertilization attempts or based on changing priorities or partner preferences. This has important implications for the use of the website and whether users will return to the tool as new decision points arise. Future studies will need to explore longitudinal website engagement and evaluate whether it meets the needs of young adult female cancer survivors who face more complicated paths to family building. The website was designed for survivors to use individually, inclusive of both single and partnered women, but exploration of the involvement of partners in decision-making processes and the need for resources is also critical [51].

Findings also indicated that the *Roadmap to Parenthood* website improved self-efficacy in managing health issues related to fertility. Participants reported that the website encouraged them to take action to manage their health and made them feel more prepared to do so. However, this may not hold true for all users, as a few participants reported neutral scores (*neither agree nor disagree*) when asked whether they felt confident and prepared to manage their concerns about family building after cancer treatment. This is consistent with qualitative feedback, in which a participant felt overwhelmed and distressed by the delivery of risk information and implications for potential challenges in family building. We have gathered strong evidence that the website made users feel more informed about their fertility and family-building options. However, a lingering question is whether women feel equipped to manage emotions that arise when facing decision-making tasks and whether they are prepared to pursue family-building goals. It may be that dissemination and implementation strategies should include health care providers, integration with survivorship care visits, and counseling for immediate added support if needed.

One of the main objectives of the decision aid tool was to make users aware of family-building options, including realistic expectations about potential difficulties, while also inspiring hope that parenthood may be achieved and that early planning may help to avoid or mitigate challenges. The delivery of information about risks and challenges may naturally be upsetting. A participant stated that the graphs depicting the effects of cancer treatment on ovarian reserve was “terrifying.” However, when asked whether images on the website were generally distressing, only 10% (1/10) of the participants agreed. Lim et al [52] noted that website development and evaluation typically focus on traditional usability aspects (eg, screen layout and navigation features), whereas the emotional experiences of users and, in turn, the ways in which a website supports users’ emotions are more likely to be neglected. For websites that deliver health information that may include distressing news, even those without personalized data, such as ours, considering the emotional impact of design features is important. Choe et al [53] put forth *hypotheses* for implementing empathic communication within digital health systems, including normalization of users’ emotional experiences (eg, “Many people feel worried or upset when learning this news...”) and helping the user to identify clear, actionable steps that can be taken (eg, “There are things you can do to help reduce your risk...”). Consistent with these recommendations, we made design changes to reduce the impact of the perceived distressing information. Experiencing negative emotions can also be useful when interacting with technology, but the line between helpful and harmful emotions may be tenuous [54]. It may be necessary to draw from digital mental health interventions (eg, stress management, affect regulation, or mindfulness-based strategies) to include in our website to provide additional support [55-58], while balancing the scope of the intervention. Our findings indicate that only a subset of users may need additional support to manage distress, suggesting that a stepped care model may be appropriate [59].

We have partnered with a website design firm, digital health researchers, and intervention developers to explore optimal digital solutions for addressing the lingering usability issues including users’ emotional experiences. Future studies will also further explore user feedback in a single-arm pilot study [60] and assess the need for additional content, website design changes, and intervention components to meet the needs of young adult female cancer survivors who are concerned about fertility and family building in posttreatment survivorship.

### Limitations

This study evaluated the usability of a web-based decision aid and planning tool for young adult female cancer survivors considering future family building. Although it has been shown that testing a product with 5 participants can uncover approximately 80% of a product’s usability issues [37], the study included a relatively small sample size, and the results may not be generalizable. Participants were also primarily White and highly educated, and further testing with women from diverse racial, ethnic, and socioeconomic backgrounds is needed. Disparities in oncofertility care have been reported based on age, socioeconomic status, access to insurance, religious factors, and gender or sexual minority identification [61]. Greater effort to engage diverse subgroups and use methodologies that lead to representative samples is needed.

### Conclusions

The *Roadmap to Parenthood* decision aid tool fills an important resource need for young adult female cancer survivors hoping to pursue parenthood in the future. The development process involved a patient-centered approach and an iterative framework for design modifications based on user experience and feedback. General feedback about the website was positive. Future studies will include additional content and design changes to optimize usability, with a particular focus on the emotional experiences of users. We will also pilot-test a decision aid intervention using the website in a longitudinal study design [60]. This will extend the focus of oncofertility research to include survivors’ fertility and family-building experiences after treatment and survivorship care needs.

## Abbreviations

eHIQ: eHealth Impact Questionnaire
PI: principal investigator
REDCap: Research Electronic Data Capture
SUS: System Usability Scale
